# IL-21 Biased Alemtuzumab Induced Chronic Antibody-Mediated Rejection Is Reversed by LFA-1 Costimulation Blockade

**DOI:** 10.3389/fimmu.2018.02323

**Published:** 2018-10-15

**Authors:** Jean Kwun, Jaeberm Park, John S. Yi, Alton B. Farris, Allan D. Kirk, Stuart J. Knechtle

**Affiliations:** ^1^Department of Surgery, Duke Transplant Center, Duke University Medical Center, Durham, NC, United States; ^2^Division of Surgical Sciences, Department of Surgery, Duke University Medical Center, Durham, NC, United States; ^3^Department of Pathology, Emory University School of Medicine, Atlanta, GA, United States

**Keywords:** IL-21, germinal center, follicular helper T cells, heart transplantation, antibody-mediated rejection

## Abstract

Despite its excellent efficacy in controlling T cell mediated acute rejection, lymphocyte depletion may promote a humoral response. While T cell repopulation after depletion has been evaluated in many aspects, the B cell response has not been fully elucidated. We tested the hypothesis that the mechanisms also involve skewed T helper phenotype after lymphocytic depletion. Post-transplant immune response was measured from alemtuzumab treated hCD52Tg cardiac allograft recipients with or without anti-LFA-1 mAb. Alemtuzumab induction promoted serum DSA, allo-B cells, and CAV in humanized CD52 transgenic (hCD52Tg) mice after heterotopic heart transplantation. Additional anti-LFA-1 mAb treatment resulted in reduced DSA (Fold increase 4.75 ± 6.9 vs. 0.7 ± 0.5; *p* < 0.01), allo-specific B cells (0.07 ± 0.06 vs. 0.006 ± 0.002 %; *p* < 0.01), neo-intimal hyperplasia (56 ± 14% vs. 23 ± 13%; *p* < 0.05), arterial disease (77.8 ± 14.2 vs. 25.8 ± 20.1%; *p* < 0.05), and fibrosis (15 ± 23.3 vs. 4.3 ± 1.65%; *p* < 0.05) in this alemtuzumab-induced chronic antibody-mediated rejection (CAMR) model. Surprisingly, elevated serum IL-21 levels in alemtuzumab-treated mice was reduced with LFA-1 blockade. In accordance with the increased serum IL-21 level, alemtuzumab treated mice showed hyperplastic germinal center (GC) development, while the supplemental anti-LFA-1 mAb significantly reduced the GC frequency and size. We report that the incomplete T cell depletion inside of the GC leads to a systemic IL-21 dominant milieu with hyperplastic GC formation and CAMR. Conventional immunosuppression, such as tacrolimus and rapamycin, failed to reverse AMR, while co-stimulation blockade with LFA-1 corrected the GC hyperplastic response. The identification of IL-21 driven chronic AMR elucidates a novel mechanism that suggests a therapeutic approach with cytolytic induction.

## Introduction

Long-term success of heart transplantation is limited by the development of coronary allograft vasculopathy (CAV), a hallmark of chronic rejection (CR) (1). Conventional immunosuppressive strategies, such as CNI inhibitors or rapamycin, that inhibit T cell—mediated rejection do not prevent CR; indeed, ~50% of patients develop biopsy evidence of CAV within 5 years after transplantation (2, 3). This is a particularly devastating statistic for pediatric transplant recipients because children with organ transplants have the greatest need for long-term graft survival. The inability of current T cell-directed immunosuppressive therapies to target humoral responses might explain their inability to suppress chronic rejection.

Recently, considerable progress has been made in understanding the relationships between B cells, alloantibody, and chronic rejection. Studies have demonstrated that levels of donor-specific antibodies (DSA) correlate most closely with chronic rejection (4–8). Yet, despite this demonstrated association of DSA and later graft loss, exact mechanisms underlying chronic antibody-mediated rejection (CAMR) remain unknown. In addition, a lack of satisfactory animal models further hampers progress toward understanding the mechanisms of CAMR.

Lymphocytolytic induction has been widely used in organ transplantation and autoimmune disease. Induction initiated prior to or concurrent with transplantation has been shown to be beneficial in reducing maintenance immunosuppression requirements after transplantation (9, 10), and in particular, alemtuzumab (Campath-1H) induction has been shown to be highly effective in preventing acute rejection (11). Following induction with alemtuzumab, regulatory T cells (Tregs) expand disproportionately during T cell repopulation (12). However, despite its excellent efficacy controlling T cell-mediated acute rejection, alemtuzumab may paradoxically promote alloantibody production (13, 14).

Growing evidences now show that a possible contribution of follicular helper T cells (Tfh) on B cell help under current immunosuppression and antibody-mediated rejection. It is also documented that agents targeting Tfh-B cell interaction reduced post-transplant humoral response (15, 16). While many aspects of T cell repopulation after cytolytic induction are understood, the Tfh and B cell response has not yet been fully elucidated. Here we report that incomplete T cell depletion inside of the germinal center (GC) leads to a systemic IL-21-dominant milieu and subsequent formation of hyperplastic GC, which results in chronic antibody-mediated rejection (CAMR). Conventional immunosuppression with tacrolimus and rapamycin failed to reverse AMR, but targeting LFA-1 corrected the GC hyperplasticity. The identification of a possible GC response and IL-21—driven CAMR elucidates a novel mechanism to understand a modern problem that suggests a therapeutic approach with cytolytic induction.

## Materials and methods

### Animal model

Male C57BL/6 (H-2^b^), 6–8 weeks of age, were purchased from The Jackson laboratory (Bar Harbor, ME). Male hCD52Tg mice (H-2^k^), 6–8 weeks of age, were originally created in the Walldman lab and were a gift of Dr. Kirk, Duke University. All mice were used and maintained in accordance with the guidelines and compliance of the Emory or Duke Institutional Animal Research Ethics Committee. All animals received 10 μg alemtuzumab (Campath-1H) in 200 ml PBS i.p., on day −2, −1, +2, and +4 of transplantation to induce T cell depletion *in vivo*. Additionally, animals were either untreated or treated with 200 μg of anti-LFA-1 mAb (mCD11a, M17/4; Bioexpress) i.p., on days 0, 2, 4, and 6 (day 0 being the day of transplantation). Heart transplantation was performed using a modification of the methods described previously (17). Histopathologic analysis was performed on paraffin-embedded sections of heart allografts removed at necropsy. Sections were stained with either H and E or Elastic trichrome and were scored blindly according to the established clinical criteria for diagnosing heart transplant rejection (18, 19).

### Flow cytometry

Cell suspensions from spleens and lymph nodes were prepared by mechanical dissociation. Cell suspensions including blood was subjected to hypotonic lysis of RBCs. Isolated cells were washed in RPMI 1640 and 10% FBS and counted. The cells were then resuspended in FACS buffer (2% FBS, 0.2% Sodium azide PBS) and were stained with Biotin, PE, FITC, PerCp, Pac Orange, Pac Blue, APC, APC-Cy7, or APC conjugated antibodies directed at mouse CD3, CD4, CD8, CD19, CD25, CD38, CD4/CD8/F4/80 (Dump), FoxP3, IgD. For, allo-specific visualization, APC-Cy7-conjugated allogeneic (H-2K^b^/D^b^) MHC tetramer and APC-conjugated syngeneic (H-2K^k^/D^k^) tetramer were applied as previously described (20). MHC monomers were generated from NIH tetramer core and tetramerized with Streptavidin-APC-Cy7 and Streptavidin-APC, respectively. For T cell flow crossmatch, recipient serum samples (1:32 dilution) were incubated with C57BL/6 donor splenocytes (1 × 10^6^). Later, FITC-conjugated anti-mouse Ig was added after washing. The T cells were stained with APC-conjugated anti-CD3. Flow cytometric analysis was performed using a BD FACS LSRII or BD Forressa and analyzed using FlowJo (Tree Star, San Carlos, CA) software.

### Histology, immunohistochemistry, and morphological analysis

The explanted hearts underwent serial sectioning (5 μm) from the midventricular level to the base. H and E stains were performed for routine examination and grading of rejection. Elastic trichrome, B220, CD3, CD4, PNA, Ki67, and IL-21 staining was performed for morphometric analyses of arterial intimal lesions as previously reported (20). Scanned images were analyzed and measured with computer-based software (Aperio Imagescope v11). The area of grafts was quantitated by tracing the bisected explanted cardiac allografts and isografts. Luminal (L) and intimal and luminal area (I + L) were traced and the areas quantitated. Intimal thickening was calculated according to the formula I/I + L and expressed as a percentage.

### Statistical analysis

Experimental results were analyzed by a GraphPad Prism (GraphPad Software 6.0a, San Diego, CA). The log-rank test for differences in graft survival and Mann-Whitney nonparametric test were used for other data. All the data are presented as mean ± SEM unless designated in figure legend. Values of *p* which were < 0.05 were considered as statistically significant.

## Results

### Chronic antibody-mediated rejection after alemtuzumab induction

**Figure 1:**
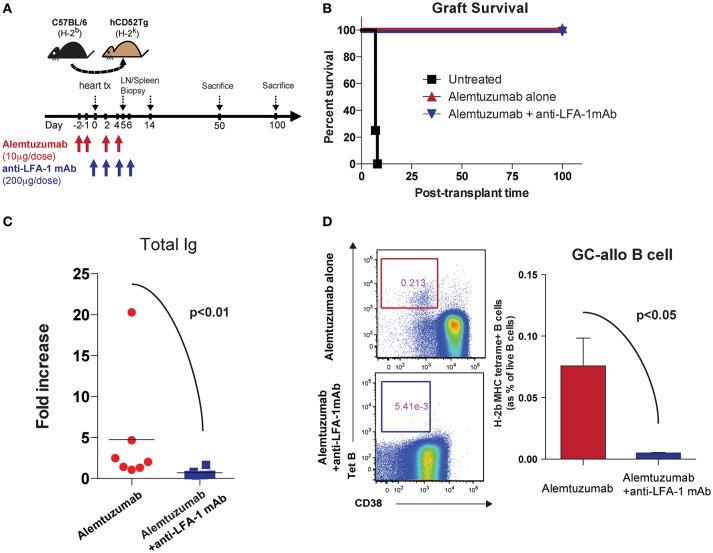
Pre-emptive anti-LFA-1 mAb treatment reduced post-transplant DSA and allo-specific B cells in alemtuzumab treated hCD52Tg cardiac allograft recipients. **(A)** Dosing scheme and experimental design. **(B)** Graft survival of human CD52Tg mice received B6 cardiac allografts. Alemtuzumab treatment (IP, 10 μg per dose at POD −2, −1, 2, 4,) with or without anti-LFA-1 mAb (KBA-1; 200 μg per dose at POD 0, 2, 4, 6) significantly prolonged graft survival (MST >100 d) vs. untreated (MST = 9 d). **(C)** Donor-specific antibody measured by T cell flow crossmatch was significantly decreased in anti-LFA-1 treatment in Alemtuzumab induced CAMR model. **(D)** Allo-specific B cells visualized by MHC (H-2K^b^/D^b^) tetramer were significantly reduced with anti-LFA-1 mAb treatment from the spleen at POD100.

We previously reported that alemtuzumab induction prevents acute rejection in humanized CD52 transgenic (hCD52Tg) mice after heterotopic heart transplantation but promotes serum DSA, allo-B cells and CAV, making this an applicable model for studying CAMR post cytolytic induction (20). Interestingly, and as is seen clinically, this heightened humoral response was not controlled by adding either tacrolimus (**Data not shown**) or rapamycin (21). We treated humanized CD52 transgenic mice with alemtuzumab with or without anti-LFA-1 mAb and monitored DSA, allospecific B (allo-B) cells and CAV development (Figure 1A). We made the surprising observation that anti-LFA-1 mAb suppressed the humoral response seen in animals treated with alemtuzumab. Anti-LFA-1 mAb treatment did not change graft survival or beating quality, which remained unpurturbed compared to alemtuzumab-alone treatment (Figure 1B). However, DSA production was greatly reduced at post-transplantation day (POD) 100 with LFA-1 blockade (Figure 1C). In addition, we tracked allo-B cells using MHC/Peptide tetramers (20). LFA-1 blockade resulted in significantly reduced allo-B cells in the spleen at POD 100 (Figure 1D). These data indicate that LFA-1 blockade prevents DSA production and suppresses allo-B cell formation, possibly by suppressing clonal B cell expansion.

### LFA-1 blockade significantly diminished chronic antibody-mediated rejection

**Figure 2:**
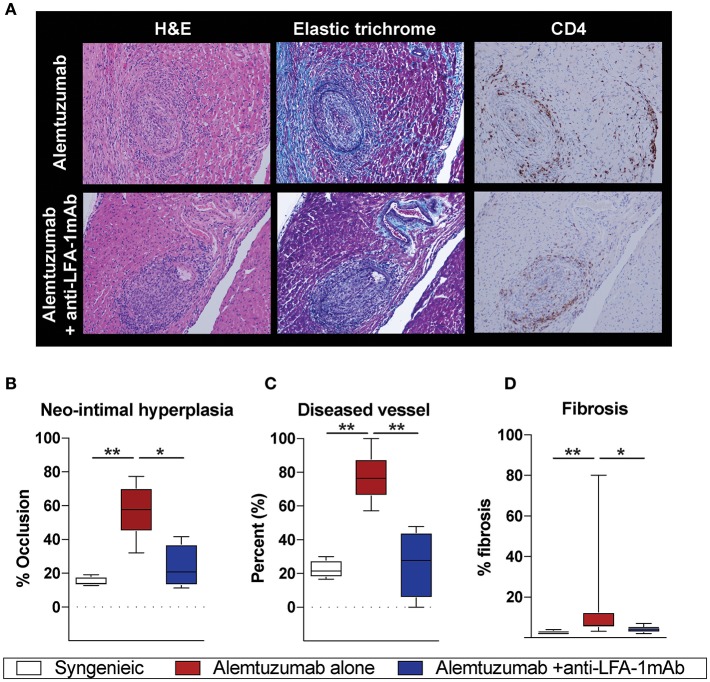
Alemtuzumab induced CAMR in human CD52 Tg cardiac allograft recipient was diminished by short-term anti-LFA-1 mAb treatment. **(A)** Representative images of H and E, Elastic trichrome, and CD4 staining in paraffin section of heart allograft from recipient showing neo-intimal hyperplasia. **(B–D)** Morphometric quantitation of lumina occlusion, occluded vessel number (over 20%), fibrosis of explanted graft from B6 heart allografts revealed reduction of CAMR with additional LFA-1 blockade. *p < 0.05, **p < 0.01.

Having observed a reduction in allo-B cells and DSA following anti-LFA-1 mAb treatment, we assessed the effect on CAV development. Cardiac coronary artery thickness was measured with Aperio scanscope program with elastic trichrome or Verhoeff staining. Even with significantly reduced DSA and allo-B cells after anti-LFA-1 mAb treatment, a noticeable amount of neo-intimal hyperplasia persisted, distinct from syngeneic controls (Figure 2A). We also noted some collapsed major coronary arteries in the anti-LFA-1 mAb–treated animals. This may represent non-DSA related CAV development. Overall, however, LFA-1 blockade significantly reduced neo-intimal hyperplasia (Figure 2B), diseased vessel number (Figure 2C), and fibrosis (Figure 2D) in the alemtuzumab-induced CAMR model. Over time, the non-functional heterotopic syngeneic cardiac allograft atrophied, likely due to the off-loaded left ventricle (22) and a limited immunologic reaction. The hypotrophic condition of allografts treated with LFA-1 blockade may represent a decreased immunologic burden when compared to allografts treated with alemtuzumab alone (Supplemental Figure 1). Collectively, we conclude that LFA-1 blockade might prevent CAV via suppression of allo-B cells in a T cell depletion—induced CAMR model.

### Systemic cytokine milieu during homeostatic T cell repopulation

**Figure 3:**
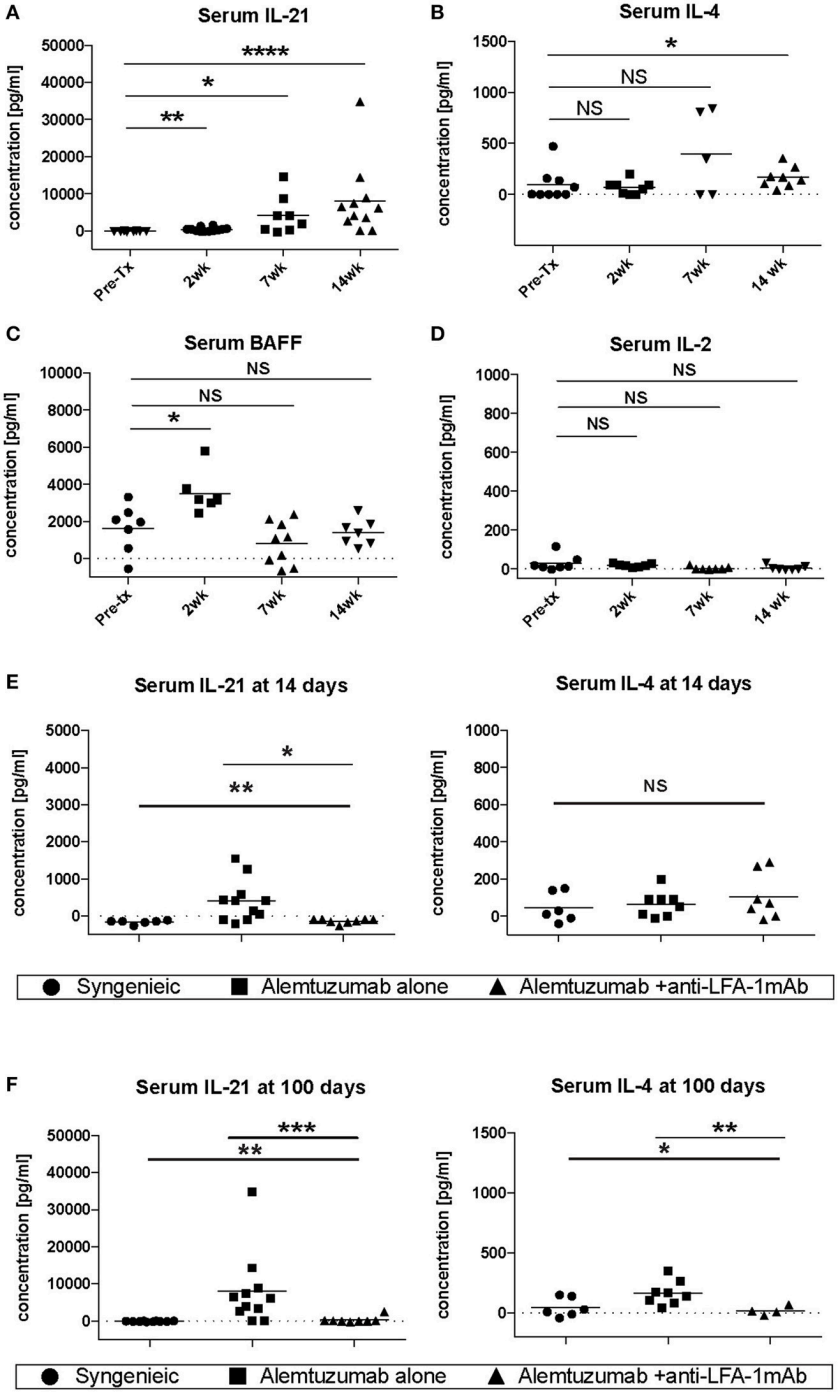
Systemic cytokine expression in human CD52Tg cardiac allograft recipients after alemtuzmab treatment. Pre- and Post-transplant level of **(A)** IL-21, **(B)** IL-4, **(C)** BAFF, and **(D)** IL-2 were measured from serum. IL-4 and IL-21 levels were significantly reduced by anti-LFA-1 mAb treatment at **(E)** Post-transplant 2 weeks and **(F)** post-transplant 14 weeks. *p < 0.05, **p < 0.01, ***p < 0.001, ****p < 0.0001.

Vascular remodeling is also affected by patterns of cytokine expression. IFN-g and other Th1 cytokines are causally implicated in stenosing vascular lesions, while Th2 cytokine expression results in abdominal aortic aneurysms (23). Many cytokines also play an important role in the control of B cell responses. IL-4 and IL-21, especially, have been shown to be important for B cell help (24, 25), and are required for an optimal humoral response (26). To address whether the decreased CAV seen during LFA-1 blockade was due to the modulation of the cytokine environment, serum IL-4, IL-21, BAFF, and IL-2 were evaluated in the absence or presence of anti-LFA-1 mAb treatment. Strikingly, IL-21 serum levels were drastically increased in alemtuzumab-treated cardiac allograft CD52Tg mouse recipients over time (Figure 3A). IL-4 levels were less impressively increased at POD 100 (Figure 3B). Serum BAFF levels were also elevated upon T cell depletion post-transplantation, similarly to what has been reported in alemtuzumab-treated human patients (27, 28). However, the serum BAFF level returned to baseline at POD 100, which might represent a transient fluctuation at an early time point, possibly due to T cell depletion—induced B cell loss (Figure 3C). Serum IL-2 levels were not changed over time after alemtuzumab treatment (Figure 3D) and suggests that only a small amount is released in the tissue. In this sense, elevated serum levels of IL-2 in CD4/CD8 mAb-treated cardiac allograft recipients might represent Th-1—driven AMR (Supplemental Figure 2). It is possible that the intensity of the Th1 response immediately following T cell depletion dictates later humoral responses. Interestingly, early and late IL-21 serum levels were significantly reduced to near-background levels following LFA-1 blockade at both time points (Figures 3E,F; *p* < 0.01). LFA-1 blockade also reduced serum IL-4 levels significantly at POD 100 (Figure 3F; *p* < 0.05). It is notable that IL-21 was not elevated in untreated (non-depleted) CD52Tg recipients at any time points, even with high levels of DSA and allo-B cells throughout the study course (data not shown), suggesting that in the face of an unopposed Th1 response in the absence of alemtuzumab treatment both IL-21 levels and the GC response is completely suppressed (29). Meanwhile, IL-4 was elevated in both untreated and alemtuzumab-treated recipients at POD 100. It is also notable that serum IL-21 and IL-4 levels were not completely suppressed by the addition of tacrolimus or rapamycin (Supplemental Figure 3). Based on the redundant yet synergistic roles of IL-4 and IL-21 in the GC response (26), insufficient suppression of either results in a robust humoral response. The segregation of IgG2a and IgG1 immunoglobulin isotypes is often used as a marker for Th1 and Th2 responses, respectively. Concordant with the response suggested by the cytokine profile, serum from untreated animals showed both IgG1 and IgG2a dominant isotypes (possibly Th1-biased), while serum from alemtuzumab treated animals showed suppression of IgG2a isotypes (Supplemental Figure 4). Reduction of IL-21 at early and late time points with LFA-1 blockade recapitulates the reduction of DSA and allo-B cell formation shown in Figure 1. These data suggest that alemtuzumab-induced T cell depletion does not induce a Th1 response, but rather promotes an IL-21—driven humoral response, thus promoting CAMR. Furthermore, our data suggests that this response might be blocked by treatment with anti-LFA-1 mAb.

### Incomplete T and GC abrogation after alemtuzumab treatment

**Figure 4:**
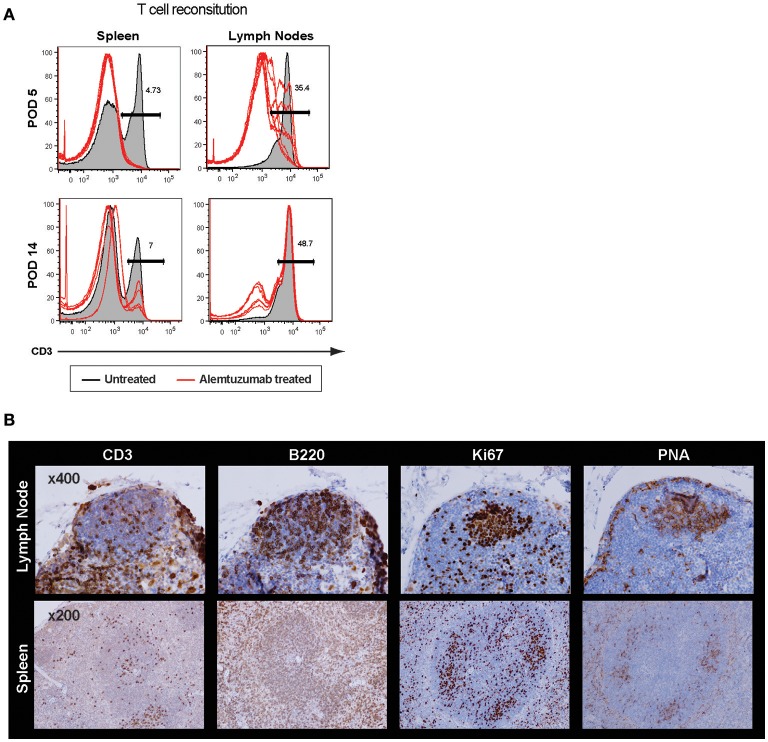
Incomplete germinal center disruption after alemtuzumab treatment in the lymph node and spleen. **(A)** Significantly higher frequency of CD3 T cells were found in the lymph nodes compared to other immune compartments at POD 5 and POD 14 (Gray area = non-depleted control, Red line = alemtuzumab treated). **(B)** Immunohistochemistry demonstrated cell components of germinal center. T, B, Ki67 and PNA staining confirmed germinal center (GC) response at 24 h after alemtuzumab treatment.

It is surprising to see rapid IL-21 production as early as 2 weeks post–T cell depletion because GC-Tfh (germinal center resident follicular helper T cells) are, theoretically, a major source of IL-21 and IL-4 production (30). Profound T cell depletion was shown in peripheral blood and spleen, but a higher number of T cells were found in lymph nodes even 24 h after T cell depletion. We accessed GC-Tfh cells *in situ* and found evidence of an intact germinal center at 2 weeks post-transplant (Supplemental Figure 5). GC structures were also found in both lymph nodes and spleen at 24 h after full alemtuzumab doses (at POD 5; Figure 4). These data are consistent with prior human data demonstrating that even when induction results in profound peripheral T cell depletion, it does not necessarily induce complete central depletion, especially for T cells inside of GC (31). It is easily speculated that GC structure (vasculature, cell components, etc.) provides some physical protection for T cells from the T cell—depleting agent. Taken together, LFA-1 blockade suppresses an IL-21 dominant/germinal center-driven, anti-donor humoral response. IL-21 production is not unique to Tfh cells; it is also produced by other T cell lineages such as Th17, Th2, and Th1 cells (32, 33). LFA-1 is expressed on both T and B cells (34), and the initial T-B cognate interaction or later interaction in the B cell follicle may be altered by anti-LFA-1 mAb. This may result in reduced development of follicular helper T cells and consequent lack of allo-B cell clonal expansion.

### Post-transplant germinal center suppression via blocking LFA-1

**Figure 5:**
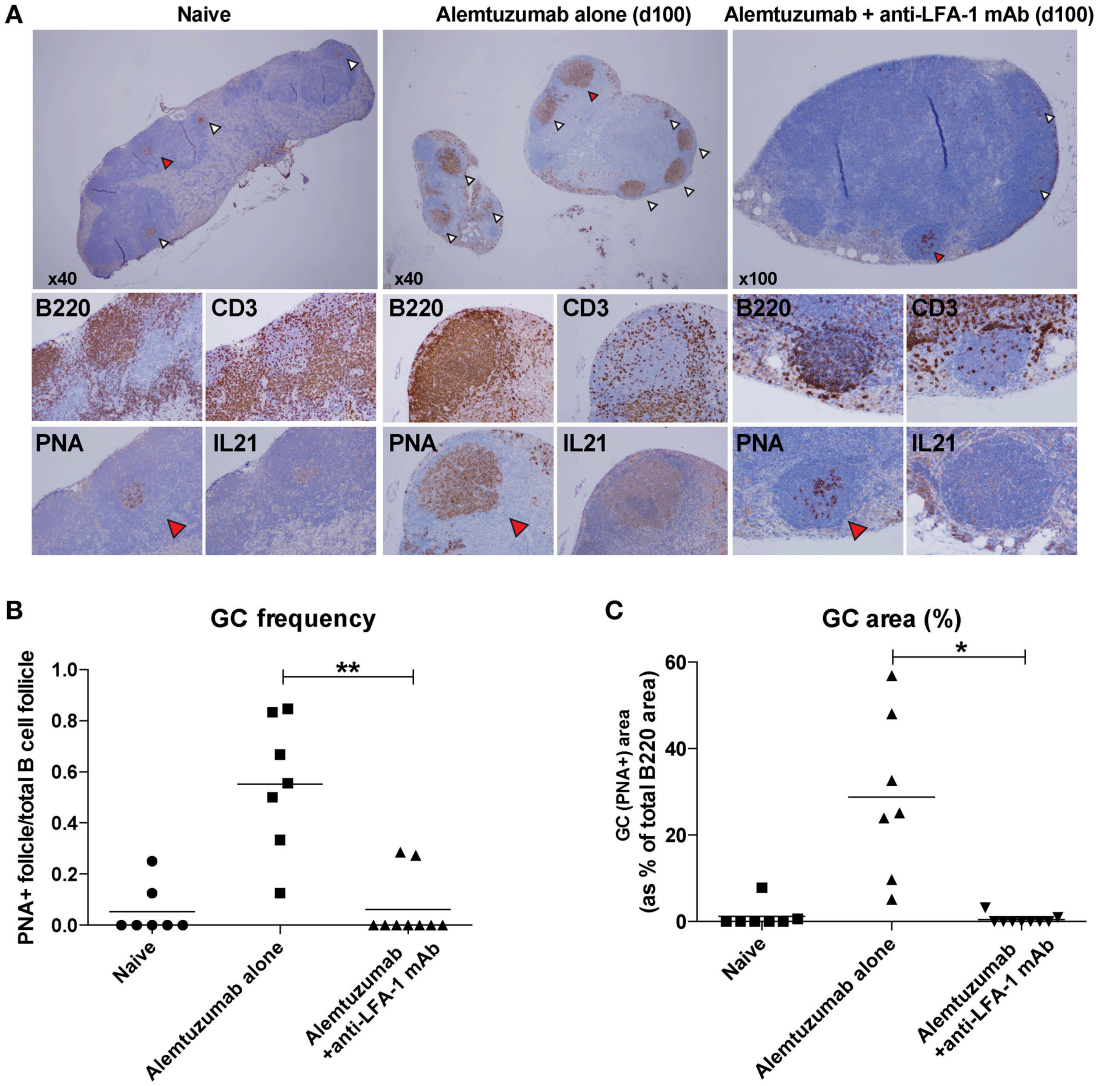
Reduction of post-transplant GC response with anti-LFA-1 mAb treatment. **(A)** post-transplant GC response was visualized by B220, CD3, PNA, and IL-21 staining *in situ*. **(B)** GC frequency was quantified by calculating the PNA^+^ follicle number by total B220+ follicle number. **(C)** GC response or hyperplasticity was measured by calculating PNA^+^ area by total B220 area. *p < 0.05, **p < 0.01.

Based on a reduction of allo-B cells and IL-21/IL-4 production following anti-LFA-1 mAb treatment, we hypothesized that LFA-1 blockade suppresses GC-Tfh cell development, B cell clonal expansion, and GC development. We evaluated GC Tfh cell responses *in situ* from lymph nodes on POD 100. Lymph nodes from naïve, alemtuzumab-alone—treated, and additional anti-LFA-1 mAb-treated recipients were stained with H and E, CD3, B220, Ki67, PNA, and IL-21. T and B cell zones were equally reconstituted in both treated groups at POD 100. Multiple hyperplastic GCs were found in samples from mice treated with alemtuzumab-alone (*n* = 7), but LFA-1 blockade reduced GC frequency and size to baseline (naïve) levels (*n* = 9). We also found that IL-21 staining was greatly reduced in the anti-LFA-1 mAb-treated group (Figure 5).

## Discussion

In transplantation, the relationship of DSA with AMR and graft outcome is a subject of much clinical study. In the clinical setting, DSA are generally detrimental to transplant outcomes via complement activation, induction of endothelial cell proliferation, and ADCC resulting in allograft dysfunction (AMR). Despite this, little is known about the mechanism by which the humoral response is induced under current clinical immunosuppression. We published a series of studies showing a possible involvement of Tfh and GC response in the secondary lymphoid organ in transplantation and antibody-mediated rejection in small and large animal models (15, 21). However, the biology of Tfh cell mediated B cell activation has not been fully elucidated in transplantation. In autoimmune diseases, unwanted Tfh dysregulation is associated with autoimmunity, and increased frequencies of peripheral Tfh cells have been reported in several autoimmune diseases, such as lupus, Sjögren syndrome, autoimmune thyroiditis, myasthenia gravis and rheumatoid arthritis (35–39). On the other hand, the expansion of Tfh cells are associated with efficacious vaccine strategies (40, 41). Autoimmune diseases are also often characterized by decreased subset of Tregs called follicular regulatory T cells (Tfr cells). Tfr cells express CXCR5 and FOXP3, and suppress B cell antibody production (42).

The hallmark cytokine produced by Tfh cells is IL-21. IL-21 is a gamma-chain cytokine with broad effects on both innate and adaptive immune responses (43). Significantly, IL-21 is required for the generation of Tfh cells (44, 45). Interestingly, IL-21 can overcome Tfr suppression to induce B cell activation, thereby demonstrating a critical role for IL-21 in dictating the germinal center response. In transplantation, it has been shown that transplant recipients with preformed DSA showed higher post-transplant circulating Tfh cell (46). Furthermore, alloantigen stimulated Tfh cell population promoted B cell differentiation to plasmablast in an IL-21 dependent manner *in vitro* (47). It is also shown the intact GC response from isolated lymphoid follicles under an immunosuppressive regimen with tacrolimus in intestinal transplant recipinet (48). Tfh and GC response were also required for chronic GvHD and inhibition of GC response greatly reduced chronic GvHD (49, 50). Collectively, the presence of IL-21 in secondary lymphoid organs or in local tertiary lymphoid structures can be detrimental to transplant tolerance due to its impact on the generation of Tfh cells, its autocrine production, and on B cell maturation and antibody production.

Unfortunately, there are no animal models demonstrating a correlation between Tfh/GC/IL-21 to the development of chronic rejection, even though, CAMR is more dependent on GC response, with critical help provided by Tfh cells. We have reported the *de novo* AMR model using alemtuzumab meditated T cell depletion with heterotopic heart transplantation (20). As follow-up studies, we tried additional immunosuppression in the model to suppress post-transplant humoral response. Counterintuitively, the addition of short-term tacrolimus (data not shown) or rapamycin (21) did not alleviate post-transplant humoral response but rather made it worse (increased DSA and AMR). As previously reported, costimulation blockade in the mouse CAMR model reversed this by reducing Tfh cells (21). In the present study, we targeted LFA-1, which is well known for their important role for T-B conjugation. We investigated the possible mechanism of CAMR by using anti-LFA-1 mAb in an Ab-dependent rejection model, independent of T cell mediated rejection.

As shown in Figure 1, additional anti-LFA-1 mAb treatment completely abolished post-transplant DSA and donor-specific B cells were strongly correlative with alleviation of AMR development. Similar to acute NHP AMR model using T cell depletion (15), CD52Tg mice with alemtuzumab showed profound circulating T cell depletion while T cells remained in the lymph nodes and spleen. We thoroughly access the location of T cells in the lymph node and spleen after T cell depletion (24 h and 7 days). It is quite surprising that the germinal centers including T cells in the B cell follicle are preferentially identified after T cell depletion (Figure 4). In accordance with this, Kirk et al. reported presence of T cells in the lymph node after alemtuzumab treatment (31). Interestingly, we also identified hyperplastic germinal center at POD100 with elevated level of DSA and AMR (Figure 5). As previously reported, T cells are fully reconstructed after alemtuzumab yet show donor-specific hyporesponsiveness (20). Based on this, the elevated level of DSA can directly cause graft injury (Figure 1). On the contrary, anti-LFA-1 mAb treated recipients showed baseline level of serum DSA, allo-specific B cells and germinal center response at POD 100 (Figures 1, 5). It is conjectured that these left over Tfh cell in the germinal center could deviate the systematic cytokine milieu that alemtuzumab treated recipients showed significantly elevated level of IL-21 in their serum (Figure 3). Taken together, the present study demonstrates that GC-dependent CAMR development after alemtuzumab treatment and anti-LFA-1 mAb treatment can prevent the development of post-transplant humoral response and CAMR.

Here, we assumed that anti-LFA-1 mAb is dissociating T-B cell conjugation during T cell repopulation, however, the impact of LFA-1 blockade cannot be limited to this since LFA-1 is not solely expressed on T and B cells (51). It is known that anti-LFA-1 mAb prevents allo-specific T cell expansion (52) and promote transplant tolerance (53–55) by destablizing T-APC conjugation as well as blocking transmigration to the graft. Therefore, anti-LFA-1 mAb can reduce Tfh cell population by suppressing general T cell activation and expansion. Nevertheless (Regardless of off target effect of anti-LFA-1 mAb), this mode of action can block the T cell help to the B cells.

Unfortunately, clinical use of anti-LFA-1 mAb is not available in clinic for organ transplantation. However, the present study showing reduction of DSA and CAMR with anti-LFA-1 mAb recapitulate the impact of costimulation blockade in large animal model and clinic (15, 16, 56). Costimulation blockade such as belatacept often show great superiority in suppressing DSA production. It is highly likely that Tfh cells are more sensitive on belatacept than other conventional immunosuppressive drugs. However, it is still not clear that anti-LFA-1 mAb and other costimulation blockades are working on pre-GC vs. post-GC status. Overall, Tfh cells are very attractive target for controlling post-transplant humoral response.

In this study, we identify a possible mechanistic pathway that regulates a *de novo* DSA response after cytolytic induction and provide a strategy for modulating the post-transplant humoral response. In particular, the ability to suppress the functional qualities of follicular helper T cells by costimulation blockade provides a new approach to induce humoral unresponsiveness in organ transplantation.

## Ethics statement

This study was carried out in accordance with the recommendations of the guidelines set forth by the National Institutes of Health and Office of Laboratory Animal Welfare. All medications and procedures were reviewed and approved by the Emory or Duke Institutional Animal Research Ethics Committee (IACUC).

## Author contributions

JK conceived the idea, designed experiments, and analyzed data. JK and JP performed mouse experiments. JK and JY performed *in vitro* experiments. AF read pathology. AK provided critical reagents and wrote the paper. JK and SK wrote the paper.

### Conflict of interest statement

The authors declare that the research was conducted in the absence of any commercial or financial relationships that could be construed as a potential conflict of interest.
