# Supplementary material for: IL-21 Biased Alemtuzumab Induced Chronic Antibody-Mediated Rejection Is Reversed by LFA-1 Costimulation Blockade
Source: Front Immunol. 2018 Oct 15;9:2323. doi: 10.3389/fimmu.2018.02323 (PMC6196291; doi:10.3389/fimmu.2018.02323)
Supplement: Supplementary file 1 [file Image_1.PDF]

Supplemental Figures

Supplemental Figure 1

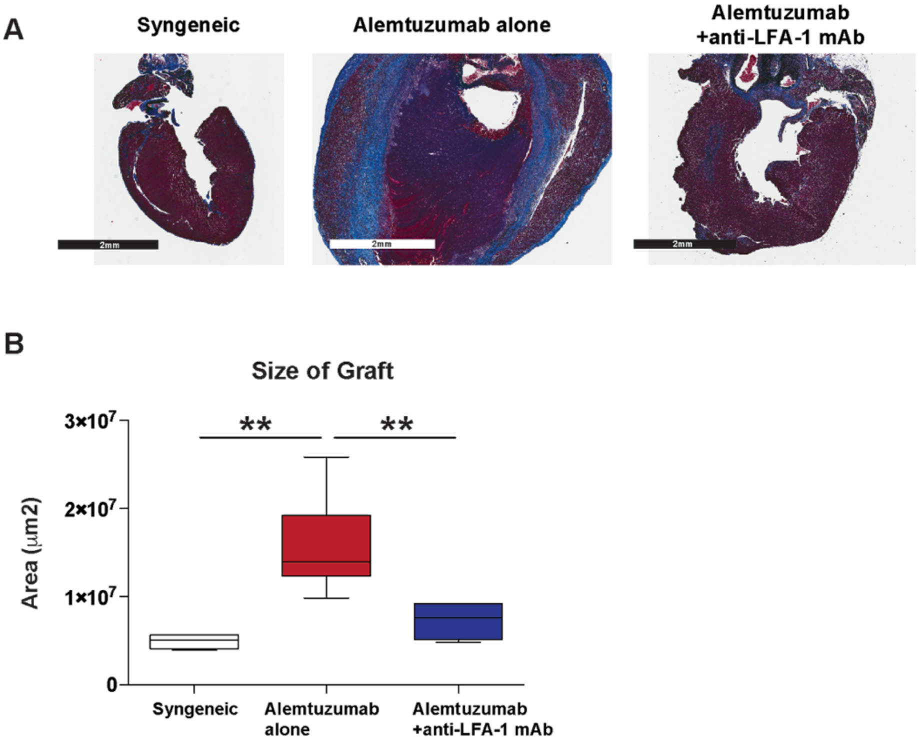

Supplemental Figure 1. Anti-LFA-1mAb treatment reduced the size of cardiac allograft compared to alemtuzumab alone treatment. (A) Representative images of explanted heart grafts from syngeneic, alemtuzumab alone treated, alemtuzumab with anti-LFA-1mAb treated recipients at 100 days after transplantation. (B) The quantified area of explanted hearts. Cardiac allografts from alemtuzumab treated recipients showed significant increase in size.

Supplemental Figure 2

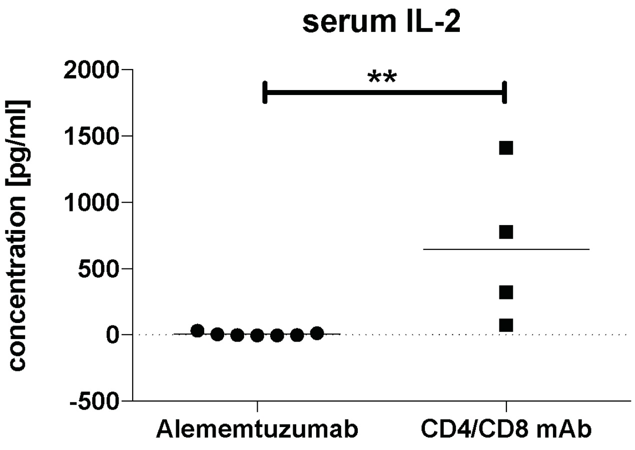

Supplemental Figure 2. Serum IL-2 expression from cardiac allograft recipients with alemtuzumab vs. anti-CD4/CD8mAbs-mediated T cell depletion.

Supplemental Figure 3

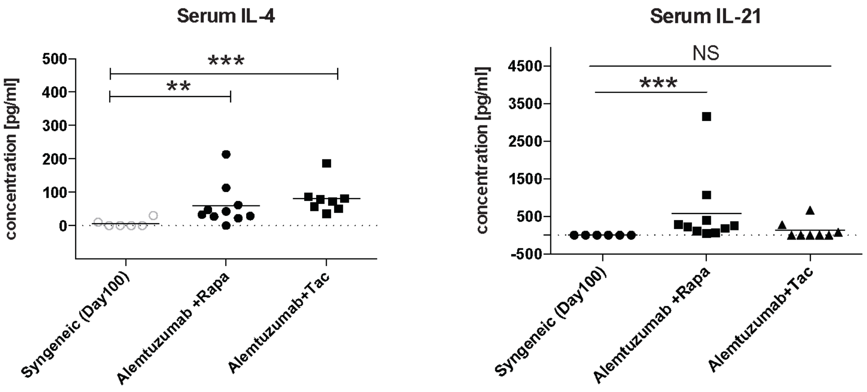

Supplemental Figure 3. The expression of serum IL-4 and IL-21 from syngeneic, alemtuzumab with tacrolimus treated, and alemtuzumab with rapamycin treated recipients at 100 days after transplantation.

Supplemental Figure 4

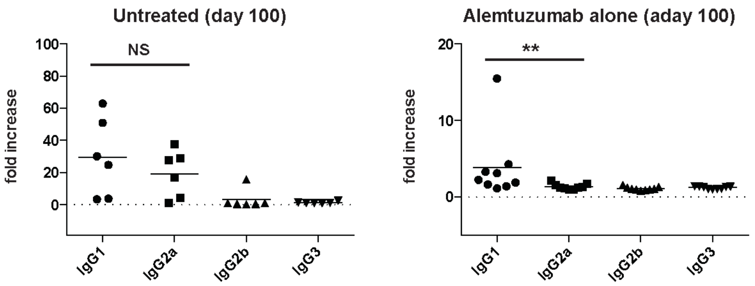

Supplemental Figure 4. Donor specific IgG antibody isotype analysis reveals that IgG2a subtype is suppressed in alemtuzumab treated recipients compared to untreated controls.

Supplemental Figure 5

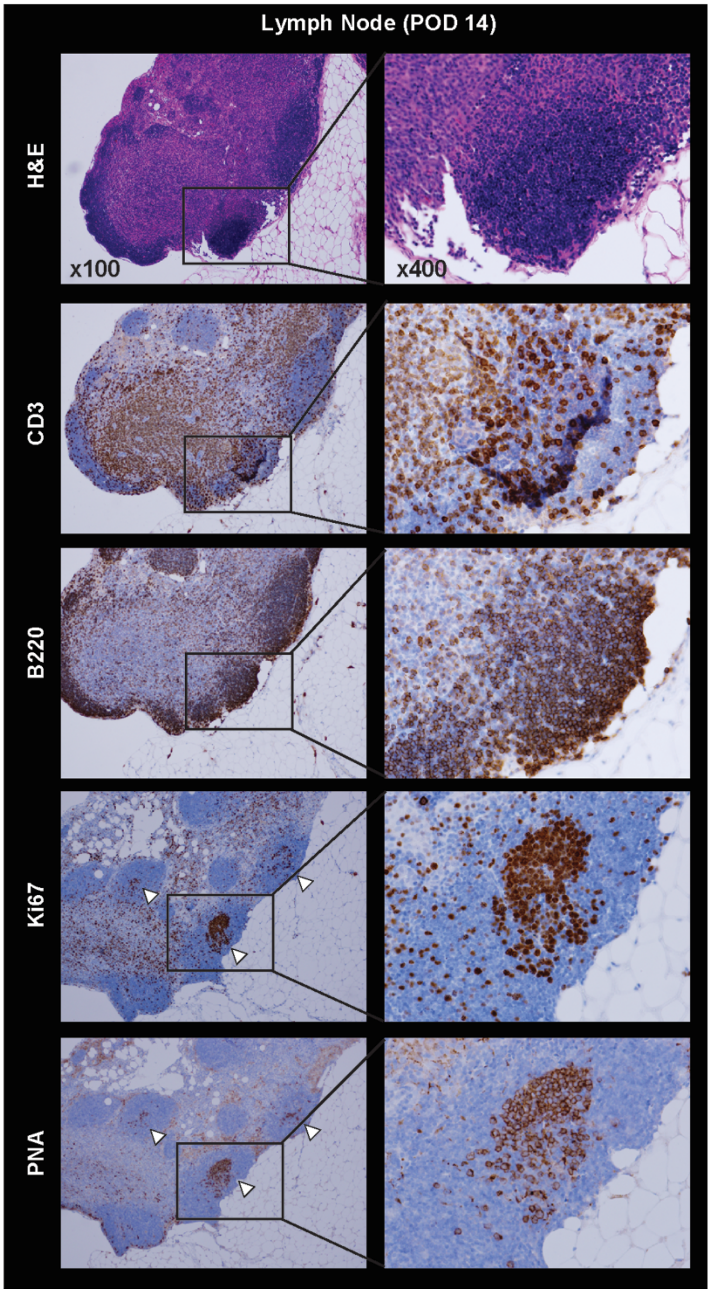

Supplemental Figure 5. Lymph node biopsy from alemtuzumab treated cardiac allograft recipients at 14 days after transplantation. White triangles (Δ) indicate germinal center response.
